# Information prescriptions: A tool for veterinary practices

**Published:** 2014-09-26

**Authors:** L.R. Kogan, R. Schoenfeld-Tacher, L. Gould, P.W. Hellyer, K. Dowers

**Affiliations:** 1 *Clinical Sciences Department, Colorado State University, Fort Collins, CO 80523, USA*; 2 *Molecular Biomedical Sciences Department, NCSU College of Veterinary Medicine, Raleigh, NC 27607, USA*

**Keywords:** Client relations, Information prescription, Internet, Private practice

## Abstract

The Internet has become a major source of health information and has the potential to offer many benefits for both human and animal health. In order for impact to be positive, however, it is critical that users be able to access reliable, trustworthy information. Although more pet owners are using the Internet to research animal health information than ever before, there remains limited research surrounding their online activities or the ability to influence owners’ online search behaviors. The current study was designed to assess the online behaviors and perceptions of pet owners after receiving either general or topic-specific information prescriptions as part of their veterinary appointment. Results indicate that nearly 60% of clients accessed the suggested websites and nearly all of these clients reported positive feelings about this addition to their veterinary services. These results suggest that offering information prescriptions to clients can facilitate better online searches by clients and positively impact both animal health and client satisfaction.

## Introduction

It is clear that the Internet has changed the way business is conducted, with veterinary medicine no exception. As of Sept 2013, 86% of U.S. adults use the Internet (Pew Research Center, 2014b) and 72% of Internet users report using the Internet for health information within the past year (Pew Research Center, 2014a).

In fields more closely related to veterinary medicine, caregivers of adults or children with significant health issues use the Internet more often for health related concerns than non-caregivers. For example, 46% of caregivers go online to research a diagnosis (compared to 28% for non-caregivers) and 72% (compared to 50% of non-caregivers) go online to gather heath information (Fox *et al.*, 2013). When health seekers go online, most use search engines; 77% report that they began their last session with a search engine (e.g., Google, Bing, Yahoo). Only 13% report starting with a specialized health information site (e.g., WebMD) (Fox and Duggan, 2013).

Although significant research has focused on Internet search behavior as it relates to human health, there has been limited research exploring how pet owners use the Internet for pet health information. Kogan *et al*. (2012) found that 13.4% of Internet users who own pets use the Internet to search for pet health information at least weekly and an additional 24.2% at least monthly. The most common reasons reported by clients for online pet health information searches are curiosity (47.4%) or the desire for clarification of information given by their veterinarian (33.6%) (Kogan *et al.*, 2010). Preliminary studies seem to suggest that pet owners view the Internet as a supplement to their veterinarian’s advice rather than a replacement (Kogan *et al.*, 2010, 2012, 2014).

When asked to compare the trustworthiness of information from different sources, clients reported the most trustworthy sources as ‘veterinarian’ (97.2% rated as trustworthy) compared to the Internet (43.5% rated as trustworthy) (Kogan *et al.*, 2010). This is similar to findings reported by Hofmeister *et al*. (2008) who found that veterinary clients rank the Internet as the third most commonly consulted source of information about pet health, behind general practitioners and veterinary specialists.

Accurate online information is important to both veterinarians and clients. A recent poll of veterinarians found that 67% reported that their clients frequently brought Internet information with them to appointments, yet 61% feel that the availability of veterinary information on the Internet confuses their clients (Fleishman-Hillard, 2008).

One way to help guide clients to accurate, appropriate pet health information is through information prescriptions. Information prescriptions were first introduced as a means for health care providers to guide patients to reliable, understandable, up-to-date information about a particular disease or condition. Often, an information prescription includes a written referral by a health care provider to a consumer health information resource (Huber *et al.*, 2012). Information prescriptions have been used to support patients’ desire to access evidence-based health information and support more informed decision making processes (Veterinary Economics, 2014). For example, a short article with an example of a sample information prescription was published recently in Veterinary Economics (Veterinary Economics, 2014).

The practice of guiding veterinary clients to Internet sites, however, is still relatively uncommon within veterinary medicine. Kogan found that nearly half (47%) of veterinarians either rarely or never suggest specific websites (Kogan *et al.*, 2012). These numbers are supported by client reports in which only 20.6% indicate they have received Internet website suggestions at least ‘sometimes’ and 39.2% report never receiving website suggestions. Yet, most veterinary clients, regardless of age, gender, or education level, report they would welcome recommendations from their veterinarian for specific websites (Kogan *et al.*, 2012).

This receptivity was tested in a study investigating the distribution of generic information prescriptions to veterinary clients in which 40% of clients who received the prescription accessed the website at least one time (Kogan *et al.*, 2014). The perceptions held by the clients regarding the information prescriptions were overwhelmingly positive.

Of the clients who reported accessing the suggested website, 86.3% reported finding it helpful and 90% reported trusting the information because it was suggested by their veterinarian. The majority of pet owners found the information useful; 87.9% reported feeling the information on the site helped them make better decisions for their pets, 89.9% felt it helped them talk to their veterinarian and 83.5% felt it added to what their veterinarian had told them. Nearly all clients (92.8%) reported feeling that receiving a webpage recommendation (information prescription) from their veterinarian was a good idea.

The following study was designed to further investigate veterinary clients’ behaviors and perceptions surrounding generic information prescriptions and to introduce and assess the impact of topic-specific information prescriptions. The positive results from the distribution of a generic information prescription were the impetus to explore clients’ receptivity towards topic-specific information prescriptions.

Because distribution of specific information prescriptions requires additional effort on the part of a veterinary team, it was deemed important to first establish the impact of a generic prescription before asking clinics to stock and distribute various information prescriptions based on specific veterinary topics. Therefore, assessment in this current study included feedback from clients in the form of a survey and informal verbal feedback from participating veterinary clinics’ staff members.

## Materials and Methods

Clients of the three participating veterinary clinics received a letter describing the informed consent process and one or more information prescriptions as part of their visit. They were subsequently surveyed on their reaction and response towards the information prescription(s).

### Participating Clinics

Participants consisted of a convenience subsample chosen from a random sample of veterinary clinics from a Western metropolitan United States area used in a previous study (Kogan *et al.*, 2014). This study included only small animal veterinary clinics because most small animal veterinarians have at least one staff member (i.e., receptionist) who checks clients in and out and oversees the completion of paperwork, and could therefore oversee the distribution of the consent forms and information prescriptions.

The three targeted veterinary clinics were asked to participate in this study for three months. Each clinic was asked to distribute 300 cover letters and consent forms to all clients until the forms were depleted. Each clinic was contacted monthly to check in, send more forms as needed and answer any questions or concerns that might have arisen. Due to the fact that the clinics varied in how consistently they distributed the cover letters and consent forms, it was not possible to track the exact percentage of clients who were asked to participate in the study but chose to decline.

All clients visiting the participating veterinary clinics were given a cover letter with a consent form explaining that the clinic was assessing several types of services offered to their clients. The form asked clients if they would be willing to complete a follow-up survey on their veterinary visit experience. The consent form asked for clients’ contact information and their preference for survey access (mail or email). The clinics faxed completed consent forms to the researchers every week; at which time the researchers either mailed or emailed the survey to the participants.

All research in this study was conducted in accordance with established protocols for the use of Human Subjects. This study was approved by the Research Integrity & Compliance Review Office at Colorado State University, with authorization 120-12H. No animals were utilized in this study.

### Information Prescription

All clients received one or more information prescriptions consisting of a handout that included the URL (universal resource locator) to a general veterinary medicine website (i.e., *www.veterinarypartner.com* or *www.healthypet.com*) or a topic-specific website (e.g., allergies, anti-inflammatory medication/pain management, ear infections, vaccinations, dental care, and weight loss/obesity) as well as several tips to help clients make informed choices about where to seek online pet health information.

All information prescriptions directed clients to either Veterinary Partner or Healthy Pet websites. Veterinary Partner is a free .com site supported by the members of VIN, the Veterinary Information Network. VIN is a membership-only community of veterinarians that does not accept advertising, giving VIN a degree of independence unusual in veterinary medicine.

All articles in Veterinary Partner have an identified author with listed credentials, a date published, and a date reviewed/revised. Many articles also have photos or illustrations, and links to support groups or more information, either on Veterinary Partner or other trusted sources of information.

Healthy Pet is a site supported by The American Animal Hospital Association (AAHA), an international association of more than 42,000 veterinary care providers who treat companion animals. AAHA is the only accrediting body for animal hospitals in the U.S. and Canada.

Internet tips (e.g., noting the author and date of online sources) were also provided to clients as part of the information prescription. The document also informed clients that health-related websites published by the U.S. government (.gov), nonprofit organizations (.org) or colleges or universities (.edu) are often the most reliable sources of health information because they are usually not supported by for-profit companies, such as drug or insurance companies.

### Assessment Survey

Individuals who completed the consent form received an assessment survey created by the authors with input from community veterinarians, pet owners, and veterinary clinicians at Colorado State University and piloted in an earlier study (Kogan *et al.*, 2014).

The survey consisted of demographic questions including age, education, gender, frequency of Internet/web usage overall and to search for pet health information. Questions pertaining to the animal’s species and reason for the visit were included, as well as questions on clients’ general experience with their veterinary visit (i.e., attitudes of staff members and veterinarian, overall rating of experience). These questions were added to the survey to provide a tangible benefit to participating clinics and were not intended to be included in analysis.

Questions pertaining to use of the information prescriptions included the number of times the client visited the website referral(s), how helpful they found the site(s), their plans for utilizing the information they found online, and their feelings about the information they accessed.

### Survey Administration

All clients who frequented the participating clinics were asked to participate; no criteria for exclusion from the study were determined; all those willing to participate in the study were eligible. All clients were offered customary veterinary service with the only addition or change being the distribution of one or more information prescriptions. In order to make this process as easy as possible for participating clinics, they were asked to distribute the information prescriptions to all clients, regardless of whether the client agreed to complete the study. Follow up surveys were only sent to clients who consented to participate in the study. In this way, clinics did not have to track who completed the consent forms, ensuring maximum compliance from participating veterinary clinics.

Clients who agreed to participate in the study (n=281) were mailed a hard copy of the survey (with a self-addressed return envelope) or emailed a link to the online survey (created with Survey Monkey). Follow up with participants was completed within one week of their veterinary visit. Descriptive statistics, chi-square, and a binary general linear model were utilized for data analysis. SPSS, version 20, was used for data analysis and statistical significance level was set at *p*<0.05.

## Results

A total of 178 clients returned the surveys, for a return rate of 63.3%. No significant differences were found, using Chi Square, between electronic survey responses and paper survey responses for any of the survey questions, so all surveys were combined for analysis.

Although clinics were asked to distribute the information prescription to all clients, clinics were at times inconsistent in distributing the information prescription, making it impossible to differentiate between clients who did not remember receiving the information prescription and those who actually did not receive it.

When asked about this inconsistency, clinic staff members indicated that during times in which they were short-staffed or extremely busy, there were times in which they forgot to distribute the information prescriptions. These reasons suggest that the information prescriptions were forgotten in a random manner and therefore, it is unlikely any unforeseen bias in distribution occurred. To account for the times when staff members forgot to distribute the information prescriptions, analysis was conducted only on those clients who reported receiving the information prescription (n=137). Not all respondents answered every demographic question, so percentages are based on answers to each question.

Questions relating to clients’ veterinary visit that did not pertain to information prescriptions were compiled and sent to each individual veterinary clinic as an incentive for participating in the study and are not reported here. Survey respondents included 30 (22.4%) males and 104 (77.6%) females (3 people did not report gender). Because only 8 (5.9%) participants were under 20 or 20-30, age categories were collapsed into: 30 or younger, 40 years old or younger (26, 13.3%), 41-50 years old (32, 23.7%), 51-60 years old (37, 27.4%) and over 60 (40, 29.6%). Education status included some high school/GED [general education diploma] (14, 10.4%), some college/vocational school (44, 32.6%), college graduate (43, 31.9%) and graduate degree (34, 25.2%) (Table 1).

**Table 1:** Participant demographics.

Gender	
Male	30 (22.4%)
Female	104 (77.6%)

Age	
30 or younger	8 (5.9%)
40 years old or younger	26 (13.3%)
41-50 years old	32 (23.7%)
51-60 years old	37 (27.4%)
Over 60	40 (29.6%)

Education status	
Some high school/GED [general education diploma]	14 (10.4%)
Some college/vocational school	44 (32.6%)
College graduate	43 (31.9%)
Graduate degree	34 (25.2%)

Clients were asked how long ago they agreed to participate in the study. Options included within the past week (35, 25.7%), the past two weeks (68, 50.0%), the past month (32, 23.5%), or over one month ago (1, 0.7%). There was no significant difference in how many times clients accessed the recommended website and how much time elapsed from when they were given the information prescription (Chi square, 15.90, df 12, p .196).

Participants were asked how frequently they accessed the Internet at home or at work. Nearly all participants accessed the Internet at least weekly (125, 95.4%). There was no statistical difference based on education (Chi square, 11.83, df 15, p .692) or gender (Chi square, 6.54, df 5, p .257). There was a significant difference based on age (Chi square, 26.68, df 15, p .031), whereby older participants reported less home and work Internet usage when compared to younger respondents.

Participants were also asked how frequently they accessed the Internet for pet health information. The number who accessed the Internet for pet health information at least weekly was 19 (14.5%), while 27 (20.6%) reported at least once a month, 73 (55.7%) less than once a month and 12 (9.2%) not at all. There was no statistical difference based on education (Chi square, 21.46, df 15, p .123) or gender (Chi square, 4.77, df 5, p .444), although there was a difference based on age (Chi square, 29.10, df 15, p .016). Older participants reported using the Internet for pet health information less often than younger respondents.

Clients were given one or more information prescriptions, driven by the reason for the visit. The information prescription form used most often was the general topic one (86, 62.8%), followed by vaccinations (49, 35.8%), dental care (30, 21.9%), anti-inflammatory medication (32, 23.4%), weight loss/obesity (19, 13.9%), allergies (14, 10.2%), and ear infections (9, 6.6%).

Clients were asked how many times they accessed any of the recommended websites they received since their veterinary visit. Nearly 60% (77) who reported receiving one or more information prescriptions indicated they had accessed the website(s). Most accessed the site(s) one time (52, 39.1%), with decreasing numbers of clients viewing it more often - twice (13, 9.8%), 3-5 times (2, 1.5%), or at least once but do not recall how many times (10, 7.5%). Fifty-six (42.1%) clients reported not visiting the website at all and 4 (2.9%) did not respond to the question. There was no significant difference in the number of times clients reported accessing the website based on gender (Chi square, 4.41, df 4, p .353), age (Chi square, 9.57, df 12, p .653) or education level (Chi square, 11.62, df 12, p .477) or how often they accessed the Internet at home or work (Chi square, 20.66, df 20, p .418).

Of the clients who reported accessing the suggested website, 72 (93.5%) reported finding it ‘very helpful’ or ‘somewhat helpful’, 5 (6.5%) neutral and no one felt it was unhelpful. When asked to indicate how they have used or plan to use the information, the most common response was “improve my understanding of an illness or health condition” (41, 53.2%), followed by “plan to look for more pet health information” (27, 35.1%); “discuss with veterinarian” (24, 31.2%); “influence future health decisions” (23, 29.9%); and “discuss with friends or family” (20, 26.0%).

Client feedback pertaining to the websites was positive. Client trust was high; 47 (61.0%) strongly agreed and 25 (32.5%) somewhat agreed that they trusted the information on the recommended site because it was suggested by their veterinarian. Most clients (69, 89.6%) reported feeling the information on the site helped them make better decisions for their pets. A significant number reported that it helped them talk to their veterinarian (66, 86.8%), and added to what their veterinarian had told them (60, 77.9%). The majority (89.6%) of clients reported feeling that receiving a webpage recommendation (information prescription) is a good idea, and 83.1% reported they plan to visit the website again in the future. The clients who did not access the website were asked to indicate all reasons for their decision. The most common reasons given included the desire to talk to their veterinarian (25, 44.6%), not having time (23, 41.1%) and forgetting (10, 17.9%) (Table 2).

**Table 2:** Reasons for not using the information prescription

Reason	Number (percentage)
Would rather talk to my vet	25 (44.6%)
I have not had time	23 (41.1%)
I forgot	10 (17.9%)
I already know enough about the medical aspects of my pet	7 (12.5%)
It is just not my nature to read about pet medical issues	5 (8.9%)
No confidence in the Internet as a source of health care information	2 (3.6%)
It is upsetting to read about an illness that affects my pet	1 (1.8%)
I do not have access to a computer and/or the Internet	1 (1.8%)
I do not use the Internet because it is too complicated	-
It is difficult for me, at times, to understand written health information	-
I prefer another Internet source for health information, rather than the websites recommended	-

## Discussion

The current study assessed the receptivity of veterinary clients to receiving either general or topic-specific information prescriptions as part of their veterinary appointment. Of those who remembered receiving an information prescription, 57.9% accessed the website at least one time with no differences based on gender, age, education level, or how often they accessed the Internet at home or work. These results are similar to an earlier study which found that when given a generic information prescription, nearly 40% of veterinary clients visited the recommended site at least once with no difference based on gender, age or education level or how often they accessed the Internet at home or work (Kogan *et al.*, 2014).

The feedback from clients who accessed any of the prescribed sites was overwhelmingly positive, for both the site and their veterinarian for making the recommendation. Most clients found the prescribed site helpful (93.5%) and planned to use it to improve their understanding of their pet’s health condition (53.2%). Clients’ trust in the recommended sites was high; with over 90% feeling they could trust the information on the recommended site because it was suggested by their veterinarian.

Most clients reported feeling a website recommendation by their veterinarian was a good idea and that they planned to re-visit the site in the future. Nearly all clients felt the site helped them make better health care decisions for their pets and facilitated better communication with their veterinarians, both by helping them talk to their veterinarian and adding to the information they were given. Feedback from the participating veterinary clinics was also positive. They reported that the amount of effort required to distribute the specific topic information prescription was minimal and they felt the effort was rewarded with more informed clients.

For those clients who did not access the recommended website, the most common reason was the desire to talk to their veterinarian instead, followed by the fact that they did not remember or lacked time. These results support previous research (Kogan *et al.*, 2010, 2012, 2014).

As the field of veterinary medicine moves towards client-centered interactions, it is important for veterinarians to acknowledge clients’ online health information searching behaviors, discuss the information offered by their clients as well as guide clients to reliable and accurate pet health websites. To help prepare veterinarians for this evolving role, courses in veterinary school curriculums such as ‘health informatics’ or ‘client informatics’ might be helpful (Kogan *et al.*, 2014). Many veterinary programs have a general course in practice management or communication in which this topic could be a natural fit.

Limitations to the current study include a limited number of veterinary clinics and some inconsistency in distributing the information prescription. The sample also consisted of more females then males, yet is representative of pet ownership and primary animal caretakers in the US. Women are more likely to own a pet (69%) than men (55%), as well as identify as the primary caretaker of the animal (81% of the time), compared to 19% of males (MarketingCharts, 2011; DeHaven, 2012). Obtaining a larger and more diverse sample of veterinary clinics, including large animal and ambulatory practices, is a possible avenue for future research.

The results of this study, however, support the idea that most veterinary clients view an information prescription favorably, thereby positively impacting the veterinary/client relationship and increasing the comfort and knowledge level of pet owners.

## Conclusion

The ease of access of online health information is dramatically changing the fields of both human and veterinary medicine. Yet, most people begin their searches with a search engine (e.g., Google), resulting in sites that vary greatly in accuracy, recency, and potential biases. Clients presenting with incorrect or misleading information can create challenges to the veterinary/client relationship as well as the health of their pet. One way to help minimize this problem is to proactively direct clients to accurate, up to date medical information.

Feedback from participants in this study, as well as previous studies suggests that clients are looking to their veterinarian for help and guidance in their online searches. Information prescriptions offer a practical, low-cost solution (Kogan *et al.*, 2012, 2014). Clients appreciate the guidance, and concurrently, become more educated, and thereby more able to partner in the health of their pet. The fear that the Internet will replace veterinary professionals was not supported in this study. Instead, clients appear to view the online material as a useful adjunct to the information given to them by their veterinarian. They also report feeling positively about their veterinarian for offering such a service.

Certainly the Internet has become a major source of health information and is viewed as having the potential to offer many benefits. Yet, for this to happen, people must be able to access reliable, trustworthy information and feel comfortable sharing it with their health care providers (Throop and Seidman, 2009). Accurate online health information can improve clients’ understanding of their pets’ medical condition and empower them to make better health decisions. It has been suggested that the increase in available online health information is playing a key role in the shift of patients/clients roles from passive recipients to more active health care consumers (Lee, 2008). Offering information prescriptions to clients facilitates this process and can positively impact animal health, client relations and clinic success.

### List of Abbreviations

URL = Universal Resource Locator.

VIN = Veterinary Information Network.

AAHA = American Animal Hospital Association.

GED = General Education Diploma.

### Conflict of Interest

The authors confirm that this article content has no conflict of interest.
